# Application and Comparison of Different Models for Quantifying the Aquatic Community in a Dam-Controlled River

**DOI:** 10.3390/ijerph20054148

**Published:** 2023-02-25

**Authors:** Jing Liu, Chao Zang, Qiting Zuo, Chunhui Han, Stefan Krause

**Affiliations:** 1 College of Water Resources, North China University of Water Resources and Electric Power, Zhengzhou 450001, China; 2 School of Geography, Earth and Environmental Sciences, University of Birmingham, Birmingham B15 2TT, UK; 3 Graduate School of Life and Environmental Sciences, University of Tsukuba, Tsukuba 305-8571, Japan; 4 School of Water Conservancy Engineering, Zhengzhou University, Zhengzhou 450001, China; 5 LEHNA - Laboratoire d’Ecologie des Hydrosystemes Naturels et Anthropises, University of Lyon, 69622 Villeurbanne, France

**Keywords:** aquatic community simulation, water ecosystem, GA-BP, modeling comparison, dam-controlled river

## Abstract

In order to develop a better model for quantifying aquatic community using environmental factors that are easy to get, we construct quantitative aquatic community models that utilize the different relationships between water environmental impact factors and aquatic biodiversity as follows: a multi-factor linear-based (MLE) model and a black box-based ‘Genetic algorithm-BP artificial neural networks’ (GA-BP) model. A comparison of the model efficiency and their outputs is conducted by applying the models to real-life cases, referring to the 49 groups of seasonal data observed over seven field sampling campaigns in Shaying River, China, and then performing model to reproduce the seasonal and inter-annual variation of the water ecological characteristics in the Huaidian (HD) site over 10 years. The results show that (1) the MLE and GA-BP models constructed in this paper are effective in quantifying aquatic communities in dam-controlled rivers; and (2) the performance of GA-BP models based on black-box relationships in predicting the aquatic community is better, more stable, and reliable; (3) reproducing the seasonal and inter-annual aquatic biodiversity in the HD site of Shaying River shows that the seasonal variation of species diversity for phytoplankton, zooplankton, and zoobenthos are inconsistent, and the inter-annual levels of diversity are low due to the negative impact of dam control. Our models can be used as a tool for aquatic community prediction and can become a contribution to showing how quantitative models in other dam-controlled rivers to assisting in dam management strategies.

## 1. Introduction

Rivers represent some of the world’s most biodiverse ecosystems [1] and provide critical ecosystem services to society and the environment [2]. Many rivers globally are dammed, and their flow is regulated for energy, water storage, irrigation, and protection against floods and are fragmented by artificial barriers to block free flow [3,4]. The number of river dams in European exceeds one million [5], in the United States at least 90,000 are over six-feet tall, and in China reaches 100,000 with a flow of more than 5 m^3^/s. While dams are generally designed to control with the purpose of stabilizing low flows and reducing peak flows for ensuring water security [6], human survival, and economic development, they also have significant ecological impacts, e.g., by impeding the flow of essential nutrients [7,8], altering thermal regimes [9,10], modifying sediment transport [11], disrupting growth cycles of aquatic organisms [12], or affecting ecosystem structure [13,14] and functions [15] along river networks, all of which cause the reduction of biodiversity of aquatic communities [16,17,18], and the more the deviation from the natural regime, the greater the loss of those ecosystems [19]. Aquatic biodiversity refers to the groups of organisms that determine the ecological status and functioning of rivers. The ecological status of a river environment is strongly affected by the composition and condition of the populations of organisms that inhabit it, known as aquatic communities, which include phytoplankton, zooplankton, and benthos. Their composition and functional status provide useful information and quantifiable indicators for the ecological health of freshwater systems [20]. Water environmental factors, including hydrodynamics and water quality, are regarded as one of the most essential determinants of the river ecosystem [21,22]. Therefore, modeling the response relationship of aquatic communities to water environmental factors is an efficient way to simulate water ecosystem features. 

As awareness of aquatic community-related water eco-environment problems increases, model quantification of the water ecosystem in rivers remains one of the most serious challenges. Many studies have been conducted on the ecological models to quantify aquatic communities [23,24], with a main focus on the uptake model [25,26], bioenergetic model [27], evolution model of aquatic organisms [28,29,30], species interactions model [31,32], and numerical models that quantify relations between aquatic densities and diversity [33]. Those types of models are currently becoming more comprehensive, allowing for the modeling and prediction of an increasing number of aquatic features and processes [34]. However, as the complexity of the model increases, the number of data samples required, the number of parameters, and the difficulty of fitting the model all increase accordingly. In addition, ecology models generally have restricted access to source code and limited model flexibility [23]. For dam-controlled rivers, many modeling approaches are applied to evaluate the changes in hydrological regime [35], water temperature [36], or water quality [37] caused by dam building or operation. However, for water ecosystems that are regularly disturbed by dams, many evaluations have been carried out by typically using weighted bio-indicators or standard test species to analyze as evaluation endpoints [38], while model techniques are mainly focused on the eco-impact of monomer (unit) or group, e.g., fish [39], macroinvertebrates [40,41], algae [42] and vegetation [43], instead of the diversity alteration of community in the biological realm. 

Although these ecological models offer numerous advantages and benefits to practices in a wide range of scales so far, they also have weaknesses, such as the model structure that is overly complicated, constrained model flexibility, and contains a significant amount of hard-to-get biotic response data (e.g., abundance, survival of aquatic organisms). Computation time may grow with the complexity of the modeling approach and implementation, and specifically, highly over-parameterized models are intrinsically data-hungry and require substantial amounts of data and information that do not necessarily exist, creating a need for parameter estimation without much background information to constrain parameter spaces. In general, obtaining biotic response data by field experiments is more expensive and time-consuming than physical and chemical water environmental factors (e.g., hydrodynamics and water quality). Therefore, new approaches and simple models that are computationally less costly and produce outputs that are easier to interpret are needed to improve predictions of how the existence and operation of dams will influence aquatic communities, helping to frame a strategy to support healthy and sustainable water ecosystem development.

An alternative to advancing this field could be through a model imputing easy-to-get physical and chemical water environmental factors to simulate aquatic community impact by the dam to overcome the limitations of previous studies. Our group previously constructed a multivariate nonlinear regression model (MNLE model) to quantify the interactive relationship between environmental factors and aquatic communities in a dam-controlled river [44]. However, the accuracy of the model is not excellent because the nonlinear regression functions cannot truthfully describe the complex interrelated processes of aquatic communities driven by environmental factors. 

The black-box model, which does not require any assumptions about the system but can frequently precisely capture all phenomena that are properly represented by data [45], can improve model accuracy without capturing complex interrelated processes. Backpropagation (BP) neural network, which is a forward learning algorithm and an error BP neural network, is one of the typical black box models successfully developed and widely used for estimating and predicting, in particular, modeling non-linear water ecosystems and the water science field. The BP neural network has substantial self-learning and nonlinear mapping capabilities [46]. However, due to limitations such as slow convergence speed, sensitivity to weight initialization, and tendency to slip into local extremes [47], using BP neural networks directly in prediction may not achieve yield satisfactory outcomes. Genetic algorithm (GA) is a stochastic search method that can be effective in optimizing the BP neural networks to overcome its inherent limitations. GA mimics the process of natural biological evolution by employing the principle of survival of the fittest to demonstrate excellent generalization capacity and good performance with higher precision [48].

Prediction models that quantify the aquatic community’s response to water environmental factors in dam-controlled rivers have shown to be helpful tools in identifying the impact of the water ecosystem by the dam, as modeling aquatic communities simply, efficiently, and economically can be a challenge. In order to develop a better model for quantifying the water ecological environment using environmental factors that are easy to get and readily available, in this study, we expanded on previous research in this field by specifically focusing on (1) analyzing the linear and black-box response between aquatic communities and water environmental factors to develop the linear regression model (MLE model) and GA-BP model, respectively; (2) comparing the models in order to find the optimal model with the best performance; (3) applying the model to reproduce the inter-annual and seasonal variations of the aquatic community of the Huaidian (HD) site in the Shaying River (SYR), China, from 2005 to 2014. This study can become a contribution to showing how quantitative models in other dam-controlled rivers can be applied to the aquatic community to help determine management actions in the dam-controlled river to better fulfill the water eco-environment preservation goal.

## 2. Materials and Methods

### 2.1. Study Area and Data Collection

**Figure 1:**
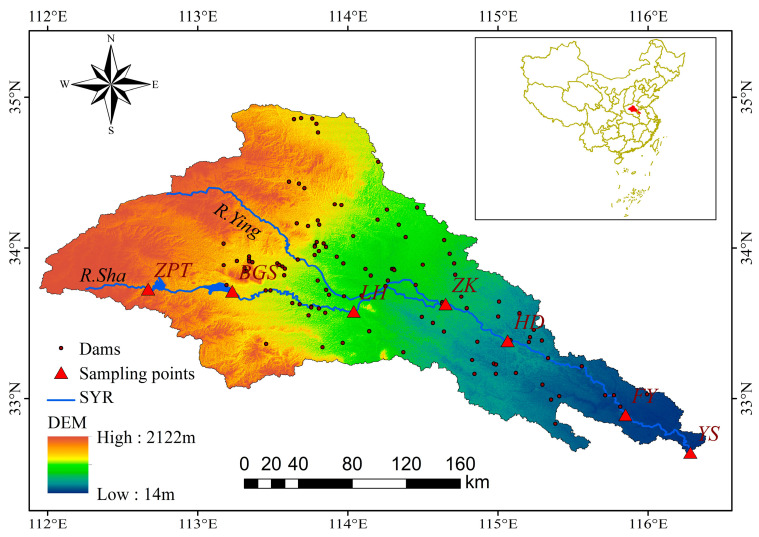
The geographical location of the research area, the topographic gradients from its sources to the outlet, and the distribution of sampling points.

The Shaying River (SYR) is the largest and perhaps most polluted tributary of the Huaihe River Basin [49], which is regulated by more than 115 dams, reservoirs, and sluices. The river flow regulation through those dams and sluices has a significant impact on the flow regime of the SYR [50], causing ecological changes [51,52]. The SYR is located between 32°31′~34°59′ of north latitude and 111°56′~116°31′ of east longitude, which belongs to the warm temperate semi-humid continental monsoon climate zone. The annual average temperature ranges from 14 °C to 16 °C, and the annual precipitation is 753.43 mm, with four distinct seasons [53]. Rainfall varies sharply during the year; the main flood season is June to August, with peak flows usually occurring in July. Forest coverage in the headwaters exceeds 80%, and cultivated land is mainly planted with wheat in the middle stream and downstream. The SYR flows through more than 40 cities and counties, which are characterized by frequent water pollution accidents and prominent contradictions in flood control and pollution prevention. Figure 1 shows the geographical location of the research area, the topographic gradients from its sources to the outlet, and the distribution of sampling points.

We divide the whole year into the dry season and the wet season. The wet season lasts from June to October, and its runoff accounts for more than 70% of the total annual runoff, while the dry season is from November to May. The SYR was selected to carry out monitoring experiments on the river ecology and environment during the dry season and the wet season to obtain seasonal data on the species and density of phytoplankton, zooplankton, and benthos. Seven sampling points (see Figure 1) were set up, and from upstream to downstream of the SYR were Zhaopingtai (ZPT), Baiguishan (BGS), Luohe (LH), Zhoukou (ZK), Huaidian (HD), Fuyang (FY), and Yingshang (YS). We carried out one field investigation in the SYR every six months to obtain the river’s ecological and environmental samples. Since December 2012, seven field sampling campaigns have been carried out, and 49 groups of seasonal data have been obtained. Both datasets of hydrodynamic and water quality are provided by the Huaihe River Commission of the Ministry of Water Resources, P.R.C., and China National Environmental Monitoring Centre (CNEMC). The seasonal water quality datasets include dissolved oxygen (*DO*), *pH*, total dissolved solids (*TDS*), ammonia nitrogen (*NH*_3_*-N*), five-day biochemical oxygen demand (*BOD*_5_), and permanganate index (*COD_Mn_*), total nitrogen (*TN*), total phosphorus (*TP*). The seasonal hydrodynamic factor includes water flow (*Q*) and temperature (*T*). In addition, habitat quality assessments were conducted for each monitoring section to determine the spatial variability of habitats in the river ecosystem [54].

### 2.2. Methodology

#### 2.2.1. Output Variables 

Aquatic biodiversity was selected as the output variable of the model. Changes in the river environment have the potential to directly cause changes to the community composition and diversity of aquatic plants and animals. In general, the more complex the community structure, the higher the biodiversity formed. The Shannon-Wiener diversity indexes based on biological data can be used to quantify the diversity of aquatic communities. The species diversity of phytoplankton (*P-SWI*), zooplankton (*Z-SWI*), and zoobenthos (*B-SWI*) were used to present the aquatic community in this research; thus, the output variables of the model are denoted as *f_P-SWI_*, *f_Z-SWI_*, and *f_B-SWI_*, respectively. The species and density of phytoplankton, zooplankton, and benthos need to be transformed into species diversity through the Shannon-Wiener index [55], and the larger the index, the higher the complexity of the community and the greater the aquatic biodiversity. The Shannon-Wiener index was calculated here as follows:

(1) $$f_{SWI}=-\sum_{i=1}^{m}p_{i}\log_{2}p_{i}$$

(2) pi=niN

where *f_SWI_* was the Shannon-Wiener index; *i* was the number of species, *i* = 1, 2, 3⋯*m*; *n_i_* was the density of the *i*-th species; *N* was the sum of the densities of all species; and *p_i_* was the proportion of the *i*-th species [56].

#### 2.2.2. Input Variables

The key factors that affect the aquatic community from the water quality and hydrodynamic factors were the input variables of the model. We obtained the key factors from our previous research, which identified the key influencing factors using Redundancy Analysis and Monte Carlo tests based on 49 groups of the seasonal water quality and hydrodynamic factors collected from the seven field sampling campaigns on the SYR, and found that *Q*, *DO*, *TP*, and *TN* were key impact factors for *f_P-SWI_*; *DO*, *Q*, and *TN* for *f_Z-SWI_*; *DO*, *Q*, and *COD_Mn_* for *f_B-SWI_* [44]. The input and output variables required for model calculation are shown in Table 1.

#### 2.2.3. MLE Model Construction

**Figure 2:**
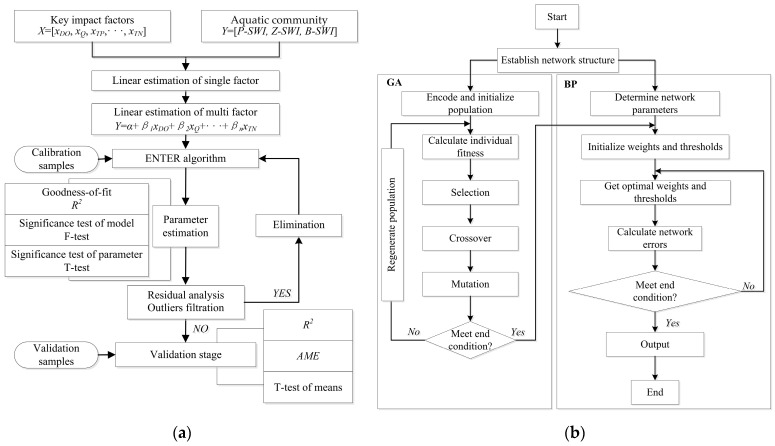
Flow diagram indicating the model conceptualization and building process of the MLE and GA-BP models. (**a**) MLE model, (**b**) GA-BP model.

The building process of the MLE model was shown in Figure 2a, which includes significance tests of the parameters for optimal correlation between output and input variables, the estimation of goodness-of-fit, residual analysis, and elimination of outliers when the residuals fall outside the range (−2, 2), etc. The specific operation flow can refer to a similar description by Zuo et al. (2019) [44]. In this paper, based on the 49 groups of data obtained from the seven monitoring experiments carried out in the SYR, 40 groups of the data (80% of total data) were randomly generated as the calibration samples, and 9 groups of the data (20% of the total data) were generated as the validation samples. The structures of the MLE models are as follows:

(3) $$f_{\mathrm{P-SWI}}=a_{P}+\beta_{P1}x_{DO}+\beta_{P2}x_{Q}+\beta_{P3}x_{TP}+\beta_{P4}x_{TN}$$

(4) $$f_{\mathrm{Z-SWI}}=\alpha_{Z}+\beta_{Z1}x_{DO}+\beta_{Z2}x_{Q}+\beta_{Z3}x_{TN}$$

(5) $$f_{\mathrm{B-SWI}}=\alpha_{B}+\beta_{B1}x_{DO}+\beta_{B2}x_{Q}+\beta_{B3}x_{COD_{Mn}}$$

where *f* is the Shannon-Wiener index/output variable; *β* is the coefficients; α_0_ is a constant; *x* is the key impact factor/input variable.

#### 2.2.4. GA-BP Model Construction

BP artificial neural networks as a member of the machine learning model, is a kind of multi-layer feedforward network trained according to an error backpropagation algorithm. One of its great advantages is that it can learn and store the mapping relationship between the input and output patterns of the network without clearly describing the mathematical relationship equation in advance. Generally, the BP neural network with a single hidden layer including enough neurons can approach any nonlinear function by adjusting its connection weight and transfer function. GA is a pseudo-biological optimization algorithm, and nature is searching for the optimal solution by simulating the evolutionary process of “survival of the fittest” for the population composed of the feasible solutions to the problem and combining natural selection and genetic phenomena. The feasible solutions are first encoded as chromosomes or individuals, and then excellent individuals with high fitness are selected for genetic operation. Genetic operation mainly includes three kinds of genetic operators: selection, crossover, and mutation. Among them, the selection and crossover operators realize the search function, and the mutation operator improves the optimization ability of the algorithm. The combination of the above two makes the model have global searchability, improves the efficiency and convergence speed, and then further improves the accuracy of the model [48]. The building process of the BP optimized by GA was shown in Figure 2b.

In this machine learning model, we constructed the BP with a three-layer network topology composed of the input layer, hidden layer, and output layer for this study. The number of neurons in the input layer was determined by the number of key impact factors, and the number of neurons in the output layer was determined by the number of the characteristic factors of the aquatic community (output variable). For the hidden layer, the determination of the number of neurons is relatively complex, and there is no mature theory at present. If the number of neurons is too small, the convergence speed and the training accuracy of the network will be low; if the number is too large, the network structure and the amount of iterative calculation will be huge and even overfitting. We used the trial-and-error method to train the network several times to determine the number of hidden layer neurons and finally obtained the best topological structure of the BP, and MATLAB was used to write the optimized BP algorithm to obtain the best solution. The initial values of neurons in the hidden layer can be obtained by the empirical formula as follows:

(6) K=2m+1

where *K* was the number of the hidden layer neurons; *m* was the number of the input layer neurons.

A Tan-Sigmoid function was chosen as the excitation function to activate neurons. To meet the range requirements of the activation function and avoid the network paralysis caused by too-large data series, the sample data should be normalized to be [−1, 1]. The normalization equation was as follows:

(7) $$y=2\;\left(\;x\;-x_{min}\;\right)\;/\;\left(x_{max}-x_{min}\;\right)\;-\;1$$

where *x* was the original data; *y* was the normalized data; *x_min_* was the minimum of the original data series; *x_max_* was the maximum of the original data series.

#### 2.2.5. Statistical Analyses

A total of 49 groups of data were obtained from the seven field sampling campaigns in SYH. The mean absolute error (*MAE*) and mean relative error (*MRE*) allowed the identification of outliers and estimation of the accuracy of the prediction models. The coefficient of determination (*R*-squared or *R*^2^) was used for correlations between predicted and observed outcomes. SPSS 22.0 was the statistical calculation software.

(8) $$MAE=\frac{1}{n}\sum_{i=1}^{n}\left|y_{i}-f_{i}\right|$$

(9) $$MRE=\frac{1}{n}\sum_{i=1}^{n}\frac{\left|y_{i}-f_{i}\right|}{y_{i}}\times 100\%$$

(10) $$R^{2}=1-\frac{\sum_{i=1}^{n}{\left(y_{i}-f_{i}\right)}^{2}}{\sum_{i=1}^{n}{\left(y_{i}-\bar{y}\right)}^{2}}$$

where *y_i_* is the true value, *f_i_* is the predictive value, and *n* is the number of predictive values. The smaller the error is and the closer *R*^2^ is to 1.0, the higher the simulation accuracy of the model is. 

The *t*-test (two-tailed) was used to compare the means of the simulation and the observation groups to test whether there was a significant difference. The null hypothesis (H_0_) is that the true difference between the two-group means is zero, and the alternate hypothesis (H_a_) is that the true difference is different from zero. *Z-score* value was confirmed by the following formula:

(11) $$\mathrm{Z-score}=\frac{{\overset{-}{x}}_{1}-{\overset{-}{x}}_{2}}{\sqrt{\left(S^{2}\left(\frac{1}{n_{1}}+\frac{1}{n_{2}}\right)\right)}}$$

where ${\bar{x}}_{1}$ and ${\bar{x}}_{2}$ are the mean values of the simulation group and observation group respectively. $S^{2}$ is pooled standard error of the two groups. $n_{1}$ and $n_{2}$ are the sample numbers. The null hypothesis is accepted if |*Z-score*| ≤ 1.96.

## 3. Results

### 3.1. Model Structure and Parameter Setting

#### 3.1.1. MLE Model

After deleting the sets of standardized residual exception data that fall outside the region (−2, 2), the “ENTER algorithm” was used to create the ideal fitting model from the randomly screened out 40 groups of sample data. According to the quantization procedure of the MLE model (Figure 2a) and the “ENTER algorithm” based on SPSS 22.0, Equations (3)~(5) can be rewritten as follows:

(12) $$f_{\mathrm{P-SWI}}=2.1809+0.1228x_{DO}+0.0043x_{Q}-0.7810x_{TP}-0.0598x_{TN}$$

(13) $$f_{\mathrm{Z-SWI}}=-0.0553+0.1894x_{DO}+0.0062x_{Q}-0.0048x_{TN}$$

(14) $$f_{\mathrm{B-SWI}}=0.5772+0.1275x_{DO}+0.0138x_{Q}-0.2294x_{COD_{Mn}}$$

Table 2 displays the results of the goodness-of-fit and F tests for the MLE models. The *R*^2^ for *P-SWI*, *Z-SWI*, and *B-SWI* is 0.561, 0.678, and 0.621, respectively, and the F tests for all of the models are significant at the *p* < 0.05 level, indicating that the MLE models for *P-SWI*, *Z-SWI*, and *B-SWI* have passed the 95% level of a significance test. The results of the t-test used to find the optimal parameters in the MLE models are shown in Table 3. Except for *B-SWI*, the majority of other models’ parameters and constant coefficients failed the 95% level of a significance test, implying that while MLE models are significant, not all key impact factors have a significant linear relationship with aquatic communities.

#### 3.1.2. GA-BP Model 

The key impact factors are used as input variables of the models. The model parameters include transfer function, training function, learning rate (*v*), training times (*epochs*), population (*Mp*), iteration times (*Ts*), crossover probability (*pc*), and mutation probability (*pm*). After several model tests based on the trial-and-error method, the best number of hidden layer neurons for *P-SWI*, *Z-SWI*, and *B-SWI* is 9, 3, and 4, respectively. The model structures and parameters were shown in Table 4.

### 3.2. Model Calibration and Validation 

#### 3.2.1. MLE Model

**Figure 3:**
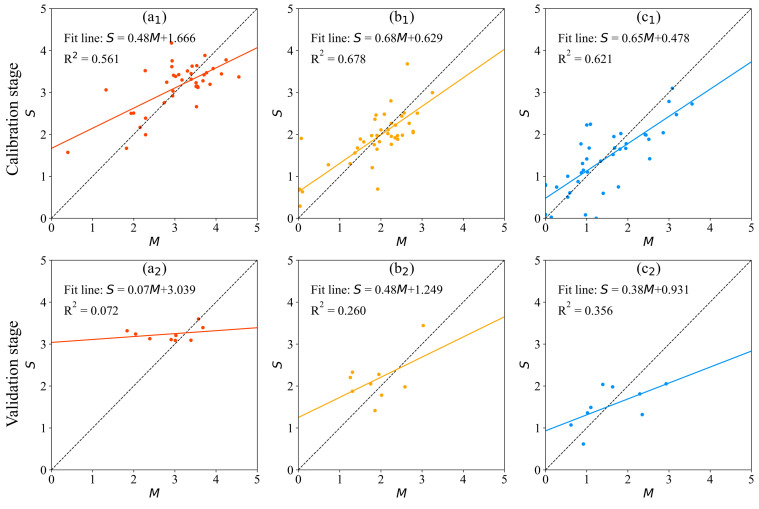
The performance of the MLE models. *M*: measured values; *S*: simulated values. Subgraphs (**a**–**c**) represent *P-SWI*, *Z-SWI*, and *B-SWI*, respectively. Subscripts 1 and 2 represent the calibration stage and the validation stage, respectively.

After the optimal parameters of the MLE models were obtained, the performance of the MLE models at the calibration and validation stage is presented in Figure 3. At the calibration stage, the mean absolute error (*MAE*) of the MLE models for *P-SWI*, *Z-SWI*, and *B-SWI* were 0.432, 0.355, and 0.525, respectively, and the *R*^2^ are 0.561, 0.678, and 0.621, respectively. At the validation stage, the *MAE* of the MLE models for *P-SWI*, *Z-SWI*, and *B-SWI* were 0.498, 0.539, and 0.599, respectively, and the *R*^2^ was 0.072, 0.260, and 0.351, respectively. It can be seen in Figure 3 that the performance of the MLE models is reasonable in the calibration stage but not ideal in the validation stage, particularly for *P-SWI*, where the fitting accuracy is the worst.

#### 3.2.2. GA-BP Model

**Figure 4:**
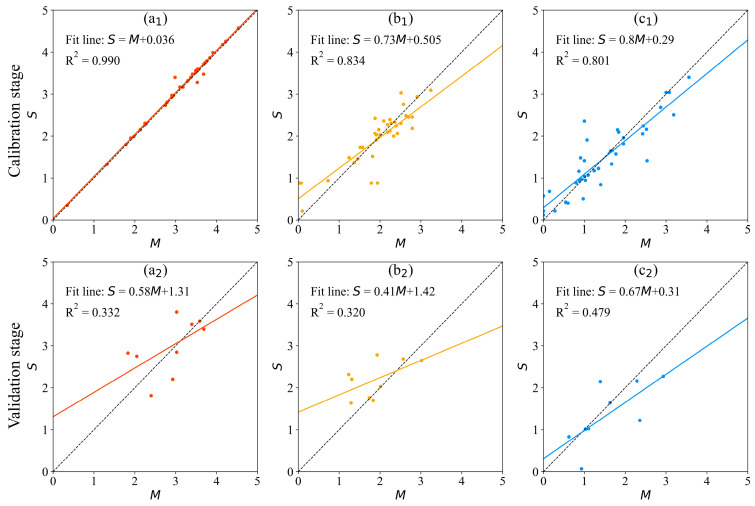
The performance of the GA-BP models. *M*: measured values; *S*: simulated values. Subgraphs (**a**–**c**) represent *P-SWI*, *Z-SWI*, and *B-SWI*, respectively. Subscripts 1 and 2 represent the calibration stage and the validation stage, respectively.

GA-BP models were established to predict the characteristics of the aquatic community. A total of 49 groups of data are selected to model the prediction model, and the numbers of calibration samples and validation samples are the same as in the MLE models. For the model of *P-SWI*, each group of sample data in the data set contains four related parameters and one original parameter, so the input matrix and output matrix of the model training sample set are 40 × 4 dimensions and 40 × 1 dimensions, respectively, and the input matrix and output matrix of the model test sample set are 9 × 4 dimensions and 9 × 1 dimension, respectively. For the models of *Z-SWI* and *B-SWI*, each group of sample data in the data set contains three related parameters and one original parameter, so the input matrix and output matrix of the model training sample set are 40 × 3 dimensions and 40 × 1 dimensions, respectively, and the input matrix and output matrix of the model test sample set are 9 × 3 dimensions and 9 × 1 dimension, respectively. Figure 4 shows the performance of the GA-BP models. At the calibration stage, the *MAE* of the GA-BP models for *P-SWI*, *Z-SWI*, and *B-SWI* were 0.051, 0.296, and 0.286, respectively, and the R^2^ was 0.990, 0.834, and 0.801, respectively. At the validation stage, The *MAE* of the GA-BP models for *P-SWI*, *Z-SWI*, and *B-SWI* were 0.486, 0.426, and 0.427, respectively, and the R^2^ was 0.332, 0.320, and 0.479, respectively.

We performed *t*-tests on the means of simulated and observed outputs to further verify the reliability and rationality of the models, and the results are shown in Table 5. Both the MLE and GA-BP models for *P-SWI*, *Z-SWI*, and *B-SWI* were not statistically different between simulation and observation at the 0.05 level, showing that the models constructed herein of *P-SWI*, *Z-SWI*, and *B-SWI* are valid. 

### 3.3. Model Comparison

The performance of the tested models is demonstrated in Table 6 by a comparison of the MLE models, MNLE models completed by our team previously [44], and GA-BP models. At the calibration stage, the performance order of the models is GA-BP model > MNLE model > MLE model. The *R*^2^ of the GA-BP models is the highest, and the *MAE* is the lowest, revealing that the GA-BP models have the best accuracy. At the validation stage, although the *R^2^* of the GA-BP model for *P-SWI* is slightly lower than that of the MNLE model, *MAE* is better. For *B-SWI* and *Z-SWI*, both *R*^2^ and *MAE* of the GA-BP models are better than that of the MLE and MNLE models. 

In addition, at the calibration stage, the *MRE* of GA-BP models for *P-SWI*, *Z-SWI*, and *B-SWI* were 1.6%, 13%, and 18.5%, respectively, which was much lower than that of MLE and MNLE models. At the validation stage, the *MRE* of GA-BP models for *P-SWI*, *Z-SWI*, and *B-SWI* increased to 20%, 28.2%, and 28.1%, respectively, but it remained the lowest when compared to the MLE and MNLE models. As a result, the simulation performance of the GA-BP models is better than that of the MLE and MNLE models and is more stable and reliable in reproducing the river ecological environment. It can be concluded that the relationship between water environment factors and aquatic community in the dam-controlled river is exceedingly complicated and cannot be explained just by linear correlations.

## 4. Discussion

### 4.1. Variability of Habitat Quality

The adaptability of the constructed model to all sampling sites in the study area is a major concern that cannot be ignored. Given the variability of habitats, directly quantifying the aquatic communities under different habitat qualities will not result in the same values each time or for each sampling site. Thus, it is important to assess the variability in habitat quality that can cause heterogeneity in aquatic communities. In this study, the habitat assessment index (*HQI*) from Wei et al. (2009) [54] was used to identify the variability of habitat quality, which included ten parameters (substrate, habitat complexity, velocity-depth combination, bank stability, bank conservation, vegetation cover, vegetation diversity, the intensity of human activities, water cognition, and riverside land use). 

**Figure 5:**
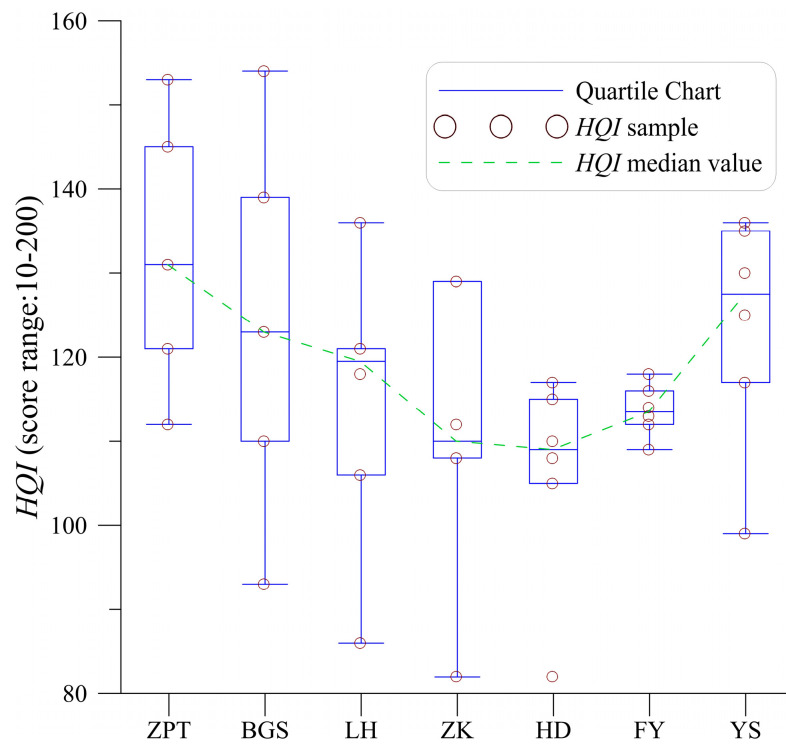
The assessment results of the habitat quality index (*HQI*).

Figure 5 shows the habitat quality assessment results for each site of the SYR from 2012. The average *HQI*s of the SYR range between 110 and 130 (the threshold range for habitat quality is 10–200), indicating that there is little spatial variability in habitat quality. Therefore, the constructed model can be applied to all sampling sites in SYR.

### 4.2. Aquatic Community Characteristics

**Figure 6:**
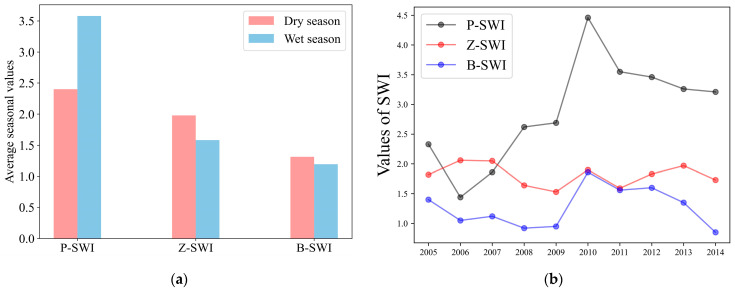
The variation of the *P-SWI*, *Z-SWI*, and *B-SWI.* (**a**) Seasonal variation. (**b**) Inter-annual variation.

The GA-BP models were applied to reproduce the aquatic community characteristics of the HD site in the SYR in the wet season and the dry season from 2005 to 2014. Figure 6a shows the ten-year average seasonal values of the *P-SWI*, *Z-SWI,* and *B-SWI*. Both in the dry season and the wet season, *P-SWI* is at the maximum, and *B-SWI* is at the minimum. For *P-SWI*, the value in the wet season is higher than that in the dry season, but for *Z-SWI* and *B-SWI*, the result is the opposite, indicating that the seasonal variation of the *P-SWI*, *Z-SWI*, and *B-SWI* are inconsistent. In natural rivers, seasonal changes of *P-SWI*, *Z-SWI*, and *B-SWI* should be consistent because the interaction of the bottom-up effect (i.e., the density, biomass, and species richness of the lower trophic class determine the population structure of the higher trophic class) and the top-down effect (i.e., the higher trophic class control and affect the community structure of the lower trophic class through predation) balances the evolution of biological community in the river ecosystem [57]. The inconsistent seasonal variation of the *P-SWI*, *Z-SWI*, and *B-SWI* in the HD site of the SYR indicates that the disturbance of dam control has a negative impact on the aquatic community of the SYR [50,52]. 

Figure 6b shows the inter-annual variation of the *P-SWI*, *Z-SWI*, and *B-SWI*. From 2005 to 2014, *P-SWI* showed a significant growth trend, *Z-SWI* changed smoothly, *B-SWI* fluctuated, but the overall trend was increasing. Generally, the growth period of phytoplankton is shorter and faster than that of zooplankton and zoobenthos, and that of zoobenthos is the slowest and the longest. Therefore, it can be inferred that the aquatic community in the HD site of the SYR has improved from 2005 to 2014. However, in addition to the values of *P-SWI* of more than 3.0, *Z-SWI* and *B-SWI* are still at a low level, especially *B-SWI*, which did not exceed 2.0 from 2005 to 2014, revealing that the ecological diversity level is still low, and the aquatic community still needs to be further repaired and protected. Perhaps dam operation based on environmental flows will be one of the effective ways to protect the disturbed-river ecosystems [58,59].

### 4.3. Model Constraints

Even while the GA-BP aquatic community model performs better than the other two models, it inevitably still has certain flaws: (1) The accuracy of the model is affected by the number of the sample database. Specifically, the more monitoring samples there are, the better the simulation accuracy of the GA-BP model. (2) The GA-BP model is a “black box” model that does not account for the physical, chemical, and biological interactions between the aquatic community and its influencing factors. (3) The model parameters cannot be directly applied to other rivers; they can only be used as a reference for modeling aquatic communities in other rivers. (4) The contribution of dam effects on the aquatic community model has yet to be quantified in the current model.

## 5. Conclusions

We provide different models of considering the actual demand to simulate the aquatic community and already present useful information about the pros and cons of the different types and structures of the models. The models are compared to real-life cases in SRY, China. The main findings of this study are as follows.

(1) MLE and GA-BP models, which we established respectively based on the linear and black-box relationships between aquatic biodiversity and water environmental factors, were effective in the simulating aquatic community in the dam-controlled river.

(2) Comparison of the MLE, MNLE, and GA-BP models, the GA-BP model performed the best in the context of this study. GA-BP model is more efficient and accurate for predicting aquatic communities by just inputting key impact factors such as water quality and hydrodynamics, which are easy to get.

(3) Applying the GA-BP model to reproduce the aquatic community characteristics of the HD site in the SYR from 2005 to 2014 revealed that the seasonal variation of the *P-SWI*, *Z-SWI*, and *B-SWI* in the HD site of the SYR was inconsistent, and the inter-annual levels of diversity were low. Dam control has a negative impact on the aquatic community, and the operation based on environmental flows will be one of the effective ways to protect the SYR’s ecosystems. 

Our models can be used as a tool for aquatic community simulation and prediction; as an economical, efficient, and referential approach in other dam-controlled rivers; and as part of water eco-environment preservation by assisting in dam planning and management strategies. The next phase of our work will focus on collecting more detailed sample data in the study area to further improve the accuracy of the model and develop physical mechanism-based models.

## Figures and Tables

**Table 1:** The input and output variables of the model.

Input Variables	Output Variables
*Q*, *DO*, *TP,* and *TN*	*f_P-SWI_*
*DO*, *Q*, and *TN*	*f_Z-SWI_*
*DO*, *Q*, and *COD_Mn_*	*f_B-SWI_*

**Table 2:** Goodness-of-fit and F tests of the MLE models.

Model	*R* ^2^	Significance Test	
		*F*	*p*
*f_P-SWI_*	0.561	10.538	0.000 *
*f_Z-SWI_*	0.678	23.903	0.000 *
*f_B-SWI_*	0.621	19.155	0.000 *

*: significance at 0.05 level.

**Table 3:** Structure and parameter settings of MEL models.

Model	Function Item	Parameter		Significance Test	
		Name	Value	*t*	*p*
*f_P-SWI_*	constant	*α* *_p_*	2.1809	4.725	0.000 *
	*x_DO_*	*β* *_p_*_1_	0.1228	3.317	0.002 *
	*x_Q_*	*β* *_p_*_2_	0.0043	1.421	0.165
	*x_TP_*	*β* *_p_*_3_	−0.7810	−0.927	0.361
	*x_TN_*	*β* *_p_*_4_	−0.0598	−1.293	0.205
*f_Z-SWI_*	constant	*α_z_*	−0.0553	−0.1650	0.870
	*x_DO_*	*β* *_z_*_1_	0.1894	7.0842	0.000 *
	*x_Q_*	*β* *_z_*_2_	0.0062	2.6388	0.013
	*x_TP_*	*β* *_z_*_3_	−0.0048	−0.1433	0.887
*f_B-SWI_*	constant	*α* *_b_*	0.5772	1.0376	0.307
	*x_DO_*	*β* *_b_*_1_	0.1275	3.2575	0.003 *
	*x_Q_*	*β* *_b_*_2_	0.0138	4.3158	0.000 *
	*x_CODMn_*	*β* *_b_*_3_	−0.2294	−2.8501	0.007 *

*: significance at 0.05 level.

**Table 4:** Structure and parameter settings of GA-BP models.

Model		GA-BP		
		*P-SWI*	*Z-SWI*	*B-SWI*
variables	input	*DO*, *Q*, *TN*, *TP*	*Q*, *DO*, *TN*	*Q*, *DO*, *COD_Mn_*
	output	*f* *_P-SWI_*	*f* *_Z-SWI_*	*f_B-SWI_*
layer nodes	input layer neurons, *m*_1_	4	3	3
	hidden layer neurons, *m*_2_	9	3	4
	output layer neurons, *m*_3_	1	1	1
parameters	transfer function	tansig and purelin		
	training function	trainlm		
	learning rate, *v*	0.1	0.1	0.1
	training times, *epochs*	100	100	50
	population, *Mp*	30	20	20
	iteration times, *Ts*	80	100	50
	crossover probability, *pc*	0.3	0.3	0.3
	mutation probability, *pm*	0.1	0.1	0.1

**Table 5:** The results of the *t*-test on the means of simulated and observed outputs.

Model	The Mean Value of Outputs		Calibration Stage			Validation Stage		
			*f* *_P-SWI_*	*f* *_Z-SWI_*	*f_B-SWI_*	*f* *_P-SWI_*	*f* *_Z-SWI_*	*f_B-SWI_*
MLE	Simulation		3.144	1.923	1.442	3.241	2.151	1.377
	Observation		3.055	1.916	1.440	2.880	1.881	1.586
	*t*-test (2-tailed)	H_0_	mean simulation = mean observation					
		*Z-score*	0.55	0.10	−0.22	1.59	0.99	−0.19
		results	no significant difference at the 0.05 level					
GA-BP	Simulation		3.082	1.908	1.442	2.971	2.195	1.377
	Observation		3.055	1.916	1.440	2.880	1.881	1.586
	*t*-test (2-tailed)	H_0_	mean simulation = mean observation					
		*Z-score*	0.14	−0.05	0.01	0.29	1.26	−0.58
		results	no significant difference at the 0.05 level					

**Table 6:** Comparison of the MLE, MNLE, and GA-BP models.

Model	Model Equation/Structure	Calibration			Validation		
		*MRE*	*MAE*	*R* ^2^	*MRE*	*MAE*	*R* ^2^
GA-BP	$f_{\mathrm{P-SWI}}:m_{1}=\;4,m_{2}=\;9,m_{3}=\;1,epochs=100,Mp=30,Ts=80$	1.6%	0.0338	0.990	20%	0.4833	0.332
	$f_{\mathrm{Z-SWI}}:m_{1}=\;3,m_{2}=\;3,m_{3}=\;1,epochs=100,Mp=20,Ts=100$	13%	0.3075	0.834	28.2%	0.4217	0.320
	$f_{\mathrm{B-SWI}}:m_{1}=\;3,m_{2}=\;4,m_{3}=\;1,epochs=50,Mp=20,Ts=50$	18.5%	0.2862	0.801	28.1%	0.4523	0.479
MLE	$f_{\mathrm{P-SWI}}=2.1809+0.1228x_{DO}+0.0043x_{Q}-0.7810x_{TP}-0.0598x_{TN}$	20.7%	0.4323	0.561	22.5%	0.4979	0.072
	$f_{\mathrm{Z-SWI}}=-0.0553+0.1894x_{DO}+0.0062x_{Q}-0.0048x_{TN}$	17%	0.3551	0.678	33.7%	0.5394	0.260
	$f_{\mathrm{B-SWI}}=0.5772+0.1275x_{DO}+0.0138x_{Q}-0.2294x_{COD_{Mn}}$	41.5%	0.5246	0.621	37.5%	0.5985	0.356
MNLE	$f_{\mathrm{P-SWI}}=-0.2371-0.0219x_{DO}^{2}-0.0491x_{TN}^{2}+0.5080x_{DO}+0.0136x_{Q}+0.4750x_{TN}-0.0045x_{Q}x_{TN}-0.1014x_{TN}x_{TP}$	9.1%	0.2309	0.811	22%	0.5136	0.357
	$f_{\mathrm{Z-SWI}}=-1.6626+0.0013x_{Q}x_{TN}+1.5803\ln x_{DO}$	15.1%	0.3443	0.723	34.5%	0.4587	0.305
	$f_{\mathrm{B-SWI}}=0.5450-0.0262x_{Q}+0.0044x_{DO}x_{Q}+0.7714/x_{COD_{Mn}}$	42%	0.4262	0.657	29.5%	0.5179	0.407

## Data Availability

The data support the findings of this study are available from the corresponding author upon reasonable request.
